# The Addition of Pumpkin Flour Impacts the Functional and Bioactive Properties of Soft Wheat Composite Flour Blends

**DOI:** 10.3390/foods14020243

**Published:** 2025-01-14

**Authors:** Durim Alija, Remigiusz Olędzki, Daniela Nikolovska Nedelkoska, Agata Wojciechowicz-Budzisz, Gafur Xhabiri, Ewa Pejcz, Eljesa Alija, Joanna Harasym

**Affiliations:** 1 Faculty of Technology and Technical Sciences Veles, University St. Kliment Ohridski-Bitola, Dimitar Vlahov 57, 1400 Veles, North Macedonia; durim.alija@uklo.edu.mk (D.A.); daniela.nedelkoska@uklo.edu.mk (D.N.N.); 2 Faculty of Food Technology and Nutrition, University of Tetova, Str. Ilinden, nn., 1200 Tetova, North Macedonia; gafur.xhabiri@unite.edu.mk (G.X.); alijaeljesa94@gmail.com (E.A.); 3 Department of Biotechnology and Food Analysis, Wroclaw University of Economics and Business, Komandorska 118/120, 53-345 Wroclaw, Poland; remigiusz.oledzki@ue.wroc.pl; 4 Adaptive Food Systems Accelerator-Science Centre, Wroclaw University of Economics and Business, 53-345 Wroclaw, Poland; agata.wojciechowicz-budzisz@ue.wroc.pl (A.W.-B.); ewa.pejcz@ue.wroc.pl (E.P.)

**Keywords:** pumpkin flour, wheat flour blends, *Curcubita maxima* Plomo, pasting properties, rheological characteristics, antioxidant activity, techno-functional properties, gel texture, colour parameters

## Abstract

Growing interest in functional food ingredients has led to the exploration of pumpkin flour as a nutritional enhancer in wheat-based products. This study investigated the impact of pumpkin flour incorporation (0–20%) on soft wheat flour blends’ technological and bioactive properties. The comprehensive analysis included granulometric distribution, techno-functional properties (WHC, WAC, WAI, WSI, SP, OAC), pasting characteristics (RVA), gel texture (TPA), rheological behaviour (frequency sweeps), colour parameters, and bioactive compounds (TPC, DPPH, ABTS) in both water and ethanol extracts. Pumpkin flour addition systematically modified blend properties, with higher fine particle content (13.26% < 80 μm), enhancing water interaction capabilities (WHC increased from 2.52 to 3.56). Pasting behaviour showed reduced peak viscosity (2444.0 mPa·s to 1859.5 mPa·s) but enhanced gel structure stability, evidenced by increased storage modulus (112.7 Pa to 1151.0 Pa) and reduced frequency dependence. Colour parameters showed progressive darkening (L* 91.00 to 84.28) and increased yellow-orange intensity (b* 10.13 to 27.13). Bioactive properties improved significantly, with TPC increasing up to 0.57 mg/1 g DM and 0.34 GAE mg/1 g DM in water and ethanol extracts, respectively, accompanied by enhanced antioxidant activity. Pumpkin flour incorporation successfully enhanced both functional and bioactive properties of wheat flour blends, with particle size distribution and water interactions serving as fundamental determinants of technological functionality, while contributing to improved nutritional value through increased bioactive compounds.

## 1. Introduction

The introduction of bioactive substances derived from vegetables to the cereal matrices of wheat is now one of the milestones in cereal food reformulation. Pumpkin is a member of the genus *Cucurbita,* belonging to the *Cucurbitaceae* family, and it is cultivated throughout the world for use as a vegetable and in medicine. Pumpkin belongs to a group of healthy foods rich in phenolic compounds, dietary fibres, carotenoids, and vitamins [1,2]. Pumpkin has been reported as one of the vegetables that contain high contents of beta-carotene, responsible for its orange colour, and it is an important dietary source of provitamin A [3,4,5].

Antioxidant properties play a role in beta-carotene development and protect immune function against the risk of acquiring chronic diseases. In addition, food products enriched with beta-carotene, such as pumpkin powder, will be a very effective way to fight vitamin A deficiency in developing countries where vitamin A deficiencies are the highest [6].

Pumpkin’s characteristics, such as its attractive colour, low-cost production, nutritional potential, and functional properties, have piqued the interest of food scientists and nutritionists for its innovative food application [7,8,9]. Pumpkin powders, made from pumpkin parts such as seed, edible flesh, and peel, as a resource of functional foods and nutraceuticals, could be effectively used in the food and pharmaceutical sectors [10]. According to Pereira et al. (2020), pumpkin flours are characterized by low water activity, high values for absorption index and water solubility, a colourimetric profile from yellow to orange, high carotenoid and fibre contents, and high values of protein digestibility, which make them desired for food application [9].

Studies show that incorporating vegetable powders enhances not only the sensory attributes of the bread but also upgrades its appearance, flavour, and aroma; in addition, it fortifies the nutritional value by adding more essential vitamins and minerals [11,12].

However, adding vegetables to wheat recipes comes with several technical challenges. Adding pumpkin and vegetables affects the dough’s rheological properties, relevant to the baked product’s elasticity and extensibility. Several studies reported that the use of pumpkin flour has a noticeable influence on wheat flour’s physical and chemical characteristics. It has been found that pumpkin flour improves the hydration properties of the dough, which is associated with increased water hydration and dough development time and even influences the dough’s stability and mixing tolerance index [13,14]. Such changes are linked to the unique content of the pumpkin flour, in which high dietary fibre content and different groups of phytochemicals can affect the behaviour of the dough during processing. Therefore, using pumpkin flour not only changes the nutritional and functional properties of the flour but also improves the overall features of the final products, including such functional features as colour, taste, and texture, making them more attractive to consumers [14]. It has been shown that using pumpkin flour in bread recipes improves flavour, colour, and moisture retention—all essential for customer acceptability [15].

Therefore, pumpkin and soft wheat flour have a multifaceted opportunity to improve baked products’ functional, nutritional, and sensory properties. Its unique characteristics, health benefits, and sustainability make pumpkin flour an ingredient of value in innovative food product development. Therefore, further research is required to optimize the formulations of these composite flours to maximize the benefits while considering consumer acceptability. The exploration will contribute to diet quality and food security and support transforming sustainable food systems.

This study aims to investigate the effects of adding pumpkin flour (with a range of 0 to 20%) on the technical and bioactive characteristics of blends with soft wheat flour. A detailed understanding of how pumpkin flour affects water interaction, pasting behaviour, gel structure stability, colour, and antioxidant properties is obtained by analyzing the granulometric distribution and other techno-functional characteristics, such as water-holding capacity (WHC), water absorption capacity (WAC), water absorption index (WAI), water solubility index (WSI), swelling power (SP), and oil absorption capacity (OAC), as well as pasting properties, gel texture, rheological behaviour, colour parameters, and bioactive compounds.

This multifaceted strategy aims to learn how pumpkin flour could act as a nutritional enhancer, raising the nutritional content and enhancing the performance of wheat-based products through improving its bioactive qualities and optimizing techno-functional features.

## 2. Materials and Methods

### 2.1. Materials and Blend Preparation

The soft wheat flour (SWF) was bread flour (Stoislaw Mill, Stoisław, Poland). The chemical composition (according to the manufacturer per 100 g) was 1.8% fat, of which 0.4% was saturated fat; 68% carbohydrates; 12% protein; 2.9% fibre; and 0.64% ash. The pumpkin flour (*Cucurbita maxima* Plomo; 1.21% fat, 8.04% protein, 45.34% carbohydrates, 39.27% total dietary fibre (soluble dietary fibre: 14.37%; insoluble dietary fibre: 24.89%), and 6.12% ash) was grown in Kamenjane, Republic of North Macedonia. The fresh pumpkin flesh was cut into small pieces, shredded, dried in a laboratory oven at 45 ± 2 °C and a relative humidity of 60–65%, and milled, resulting in powder with a moisture content below 10%.

### 2.2. Flour Blend Preparation

A rotating drum mixer (TM100, Vevor, Guangzhou, China) with variable direction of rotation was used to make flour blends (blends of 5%, 10%, 15%, and 20% pumpkin flour and 95%, 90%, 85%, and 80% SWF) which were run for 10 min; 100% pumpkin flour was used as “PP” in the analysis, whereas 100% SWF served as the “control sample”. According to the AACC 44-19.01 method, each sample’s moisture content was 10.8% for 100% SWF; 9.76%, 9.89%, 9.31%, and 9.43% for 5%, 10%, 15%, and 20%; and 9.44% for 100% pumpkin flour.

### 2.3. Particle Size Distribution—Granulometric Analysis

Granulometric characteristics of flour samples (wheat, pumpkin and blends) were determined using the AACC 66-20.01 method, with slight modification. For determination, each of the samples (50 g) was sieved with an LPzE-2e vibratory sieve shaker (Multiserw Morek, Brzeznica, Poland) in the range of Ø0.080–200 mm, in vibration amplitude for 10 min. The % versus the initial sample weight was used to determine the percentage of each sample size. The samples were measured in triplicate.

### 2.4. Techno-Functional Properties of Blends

Water-holding capacity (WHC) is the amount of water a sample retains [16]. The parameter shows how the samples absorb water when gravity is the only force acting on them [17]. WHC was determined according to Carpentieri et al. (2024) with certain modifications [17]. Briefly, 10 mL of distilled water was added to 0.5 ± 0.005 g of sample (W_s_). After 24 h, non-absorbed water was gently removed, and the weight of the sample was checked (W_hs_). WHC (expressed as g of water held per g of sample dry matter) was calculated according to Equation (1). The analysis for each sample was carried out in triplicate.

(1) $$WHC=\frac{Whs}{Ws}$$

The gel-forming properties of flour were evaluated through the water absorption index (WAI), water solubility index (WSI), and swelling power (SP), with the method proposed by Nedviha et al. (2024) with certain modifications [18]. WAI indicates how the samples gelatinize in the cooling–heating cycle, and WSI reflects the number of compounds released from the gel into excess water. Flours’ swelling power (SP) refers to their ability to absorb water and increase volume. It is an important property that affects the quality and functionality of flour in various applications. In particular, 15 mL of distilled water was used to suspend 0.5 ± 0.005 (W_s_) of the sample in centrifuge tubes (Wt), and the tubes were then heated to 90 °C for 10 min in a water bath (MLL147, AJL Electronics, Kraków, Poland). Once the tubes had cooled to ambient temperature, they were centrifuged for 10 min at 3000× *g*. The solid content (W_ss_) was measured by weighing the sediment (W_ws_), pouring the supernatant onto a stainless-steel Petri dish (W_pd_) that had been previously weighed, and heating it in an oven (SML, Zalmed, Łomianki, Poland) at 110 °C for 24 h. Equations (2)–(4) were used to determine the WAI (g of water/g of DM), WSI (g of water/100 g of DM), and SP (g of water per g of DM). The analysis for each sample was carried out in triplicate.

WAI = W_ws_ − W_t_W_s_ (2)

WSI = W_ss_ − W_pd_W_s_ × 100WSI = W_ss_ − W_pd_W_s_ × 100 (3)

SP = W_ws_ − W_t_W_s_ × (W_ss_ − W_pd_)SP = W_ws_ − W_t_W_s_ × (W_ss_ − W_pd_) (4)

Water absorption capacity (WAC) reveals how samples absorb and retain water when the forces present during mixing and centrifugation are applied; the oil absorption capacity (OAC) and hydrophilic/lipophilic index (HLI) of the samples were determined according to the method described by Carpentieri et al. (2024), with certain modifications [17]. Briefly, 15 mL of distilled water (for WAC) or oil (for OAC) was combined with 0.5 ± 0.005 g of sample (W_s_) in centrifuge tubes (W_t_). Again, after 30 s of vortexing (Vortex 06-MX-S, Stargard, Poland), the samples were allowed to rest for 10 min before the process was repeated. The tubes were weighed (W_ts_) after the supernatants were disposed of after centrifugation for 25 min at 3000× *g* (MPW-350, MPW, Warsaw, Poland), and the remaining liquid was dried in an oven (Vindon Scientific, Rochdale, UK) at 50 °C for 25 min. Equations (5) and (6) were used for calculating WAC (g of water/g of DM), OAC (g of oil/g of DM), and HLI. The analysis for each sample was carried out in triplicate.

WAC/OAC = W_ts_ − W_t_W_s_WAC/OAC = W_ts_ − W_t_W_s_ (5)

HLI = WAC/OAC(6)

### 2.5. Pasting Properties

The pasting characteristics of the blends were assessed using the Rapid Visco Analyser StarchMaster2 (Newport Scientific, Sydney, Australia); thus, 2.5 g of the sample was transferred to an RVA container, and 28.5 g of the total weight was adjusted by adding distilled water as a solvent. The temperature profile implemented in all of the blends was as follows: maintained at 50 °C for 2 min; a heating ramp from 50 to 95 °C at a rate of 5 °C/min; maintained at 95 °C for 5 min; then cooled back to 50 °C at a rate of 5 °C/min; and lastly maintained at 50 °C for 4 min. The primary pasting characteristics measured were peak viscosity (PV), hold viscosity (TV), final viscosity (FV), breakdown (BD), and setback (SB). The analysis for each sample was carried out in triplicate.

### 2.6. Gel Texture

Gels were prepared based on the paste obtained from the procedure outlined in Section 2.5, which was used to create the gels. The resulting paste was moulded into 20 mm diameter cylindrical moulds and kept at 4 °C for 24 h. After demolding, the gels were adjusted to 20 mm in height and analyzed with a total profile analysis (TPA) test on texture analyzer FC200STAV500/300 (AXIS, Gdansk, Poland). Data were acquired and calculated using AXIS FM v.2_18 software. The analysis for each sample was carried out sixfold.

### 2.7. Frequency and Amplitude Sweep

Samples for the measurement of rheological properties were prepared using a method described in Section 2.5. Briefly, the paste obtained after the heating–cooling cycle was placed in 3 mL plastic containers and transferred to the rheometer plate after 10 min rest. Using a parallel plate geometry (40 mm diameter) of serrated steel surfaces with a 1 mm working gap and a temperature set at 25 °C, managed by a KNX2002 thermal controller, dynamic oscillatory tests of the blends were conducted using an Anton Paar MC102 rheometer (Anton Paar, Stuttgart, Germany). Using a frequency sweep conducted from 10 to 1 Hz in the linear viscoelastic area at a constant stress of 1 Pa, viscoelastic behaviour was represented in terms of the storage or elastic modulus (G′) and the loss or viscous modulus (G″). Testing points were adjusted to a power law, which allowed the calculation of a, b, and c coefficients. The analysis for each sample was carried out sixfold.

### 2.8. Colour Measurement

An RS232 serial interface on a personal computer was used to link a Konica Minolta CR-310 chroma meter (Ramsey, NJ, USA) to a data processor (DP-301) to measure colour. The CR-A50 attachment for granular sample measurement was used to measure the blend’s colour. Each value was obtained eightfold. The parameters were C* (Chroma), h**º** (hue angle), L*, a*, and b*.

### 2.9. Extract Preparation

In total, 1 g of each blend (control, 5%, 10%, 15%, and 20%) was extracted with a 10 mL ethanol (99.99%) and distilled water mixture for one hour on a radial stirrer (MX-RD PRO, ChemLand, Stargard, Poland) at 60 rotations per minute, followed by centrifugation (MPW-350, MPW, Warsaw, Poland) for 15 min at 10,000 rpm. Following centrifugation, the supernatant was analyzed for the presence of bioactive compounds (TPC), reduction potential (FRAP), total antioxidant potential (DPPH, ABTS) and reducing sugar content (DNS). A duplicate extract from a sample was obtained for each analysis.

### 2.10. Determination of Total Phenolic Compounds

The Folin–Ciocalteu reagent was used to quantify the total concentration of polyphenolic compounds of the blend’s samples using spectrophotometry (SEMCO, S91 E, Gdynia, Poland) by the method of Yen et al. (1995) with a minor adjustment [19]. To achieve this, 1.58 mL of H_2_O and 0.1 mL of the Folin–Ciocalteu reagent were added to the blend’s extracts (0.02 mL). Then, 0.3 mL of a saturated sodium carbonate solution (Na_2_CO_3_) was added following a 5 min incubation period. The total phenolic compounds were measured during a 20 min dark incubation at 38 °C. The Folin–Ciocalteu reagent creates a blue complex with a maximum absorbance of 765 nm when combined with polyphenolic compounds. For gallic acid, a standard curve was created. A duplicate analysis was performed on each sample. The calibration curve was used to determine the number of polyphenolic chemicals in the tested sample, which was then expressed in milligrams of gallic acid equivalent (GEA) per gram of dry basis (dry matter weight)—DM.

### 2.11. Determination of Antioxidant and Oxidoreductive Activities

#### 2.11.1. DPPH Assay

Using the method of Klymenko et al. in 2019 [20] with minor adjustments, the antioxidant capacity (regarding the stability of 2,2-diphenyl-1-picrylhydrazyl radical (DPPH•)) for the blend’s samples was determined spectrophotometrically (SEMCO, S91 E, Poland). To achieve this, 1 mL of a methanolic DPPH solution (0.1 mM) was mixed with 0.035 mL of the sample solution, which had been measured. The absorbance at 517 nm was measured after the mixture was stirred and allowed to sit at room temperature for 20 min. A duplicate analysis of every sample was conducted. The results were expressed in milligrams of Trolox equivalent (TE) per gram of dry matter (DM).

#### 2.11.2. ABTS Assay

The method of Sridhar et al. in 2019, with minor adjustments, was used to spectrophotometrically assess the blend’s samples’ antiradical capability against the cationic radical 2,2-azo-bis(3-ethylbenzothiazoline-6-sulfonic acid (ABTS•+) (SEMCO, S91 E, Poland) [21]; 7 mM of ABTS stock solution and 2.45 mM of potassium persulfate solution were combined to create the ABTS•+ solution, which was then incubated for 16–24 h at room temperature (23 °C) in the dark. For testing, 0.02 mL of the blend’s extract was added to 1.0 mL of the diluted ABTS•+ solution. Then, 10 s after combining the test extract with the ABTS•+ solution, the absorbance at 734 nm was measured. A duplicate analysis of every sample was conducted. The results were expressed in milligrams of Trolox equivalent (TE) per gram of dry matter (DM).

#### 2.11.3. FRAP Assay

The method of Re et al. (1999), with minor adjustments, was used to assess the reducing power (ability to reduce ferric ions, Fe^3+^) of the blend’s samples, using a spectrophotometer (SEMCO, S91 E, Poland) [22]. The blend’s extract was mixed with 1 mL of FRAP solution (300 µM acetate buffer, pH 3.6), 10 µM TPTZ in 40 µM HCl, and 20 µM FeCl3 in a 10:1:1 (*v*/*v*) ratio. The absorbance at 593 nm was measured after the mixture was stirred and allowed to stand at room temperature for 20 min. A duplicate analysis of every sample was conducted. The results were expressed in milligrams of iron (II) sulphate equivalent FeSO_4_·7H_2_O per 1 g dry matter (DM).

### 2.12. Determination of Reducing Sugar Content

By utilizing the reducing abilities of sugars towards 3,5-dinitrosalicylic acid (DNS), the sugar content of extracts for the blend’s samples was determined spectrophotometrically (SEMCO, S91 E, Poland) using a modified approach as described by Miller et al. in 1959 [23]. In total, 1 mL of DNS reagent was added to 1 mL of the blend’s sample, and everything was well combined. After that, the mixture was heated for 5 min in boiling water. The mixture’s absorbance at 535 nm was measured once it had cooled to room temperature. A duplicate analysis of every sample was conducted. The results were expressed in milligrams of glucose equivalent per gram of dry matter (DM).

### 2.13. Statistical Analyses

The variance (ANOVA) analysis of the results was evaluated with Statgraphics Centurion software (Centurion XVII.I version, StatPoint Technologies, Inc., Warrenton, VA, USA). ANOVA was performed with a previous normality of checked data using a *p*-value < 0.05 significance level.

## 3. Results and Discussion

### 3.1. Granulometric Analysis and Functional Characteristic

The granulometry of soft wheat (SWF) and pumpkin (PF) flours is shown in Table 1.

The granulometric distribution data presented in Table 1 provide insights into soft wheat flour (SWF) particle size characteristics and pumpkin flour (PF). The analysis reveals that SWF exhibits a predominant fraction of particles larger than 200 μm, accounting for 72.96% ± 10.94 of the total distribution. This is consistent with typical industrial milling practices for SWF production. In the intermediate size ranges between 180 and 106 μm, SWF shows a relatively uniform distribution pattern, with percentages ranging between 4% and 8%. Notably, the finest fraction, consisting of particles smaller than 80 μm, represents merely 1.10% ± 0.17 of the total distribution.

Meanwhile, the PF demonstrates a somewhat different distribution pattern, though it shares some similarities with SWF. While it also shows a majority of particles larger than 200 μm (60.76% ± 9.11), this percentage is notably lower than in SWF. The intermediate particle size ranges in PF, from 200 to 80 μm, display a more consistent distribution than SWF, with values consistently hovering around 4–5%. The most striking difference lies in the fine particle fraction, where PF contains a significantly higher proportion (13.26% ± 1.99) of particles smaller than 80 μm.

These distribution patterns are particularly significant as they influence functional properties. The higher proportion of fine particles in PF suggests an increased surface area for potential water interactions. The observed bimodal distribution in PF, characterized by substantial amounts in the most significant and minor fractions, likely affects particle packing density and consequently influences the functional properties of flour blends.

Such characteristics directly affect technological functionality, particularly regarding water absorption capacity, mixing properties, and the textural attributes of final products. Other researchers also observed similar results of granulometric analysis [24], where larger particles from PF can contribute to a more robust dough structure while smaller particles can enhance the overall cohesiveness of the blend.

The techno-functional characteristics of composite blends are presented in Table 2.

The data presented in Table 2 reveal comprehensive insights into the water and oil interaction capabilities of PF and soft wheat binary blends across various concentrations. In the control sample (pure SWF), we observe baseline values that serve as reference points: a water-holding capacity (WHC) of 2.52, water absorption capacity (WAC) of 1.83, and water absorption index (WAI) of 5.62, all expressed in grams of water per gram of dry matter. These values reflect the typical hydration properties of SWF, primarily influenced by its protein content and starch characteristics.

As PF concentration increases from 5% to 20%, we observe a progressive enhancement in water interaction capabilities. The WHC steadily increases from 2.52 to 3.56, indicating an enhanced ability to retain water under gravitational forces. The same trend was observed in the research of Aljahani (2022) [25]. Similarly, WAC values increase from 1.90 to 2.24, suggesting improved water binding under external forces. This trend continues across other hydration parameters, with the water solubility index (WSI) showing a notable increase from 3.80 to 8.60 g H_2_O/100 g DM as PF content rises.

Adubofuor et al. [26], Hoxha et al. [27], Pasha et al. [28] and Van Toan et al. [29] reported a similar trend in water absorption. Likewise, Eke-Ejiofor et al. (2021) [30] observed comparable results for wheat and PF blends regarding WAC and OAC. The rise in WAC observed with increasing PF ratios can be attributed to the high fibre content of the PF. The increased WSI is due to the higher fraction of small particles in PF than in SWF. The decreasing particle size may be attributed to the greater specific surface area, resulting in higher leaching of soluble starch-derived molecules dissolved in water [31]. The most dramatic differences become apparent when examining pure PF, which exhibits remarkably higher values across all parameters: WHC of 10.89, WAC of 8.01, WAI 8.64, and WSI of 29.27. Mittal et al. [32] noted much lower WAC (2.56). A similar WSI value was recorded by Saeleaw and Schleining (2011) [33] and much lower by Pereira at al. [10]. However, similar results for WAI were obtained in the study by Pereira et al. [9]. These elevated values likely stem from the higher fibre content and protein composition of PF. The high WHC value indicates that PF could be suitable for applications requiring viscosity enhancement, adequate hydration, and freshness preservation, particularly in cooked food products. Aktaş and Gerçekaslan (2024) [34] reported a higher WHC value (12.91 ± 0.40) for pumpkin pulp flour, which was close to the value obtained from passion albedo fibre. The oil absorption capacity (OAC) also consistently increases with higher PF content, ranging from 1.14 in control to 1.78 g Oil/g DM in pure PF, suggesting enhanced lipophilic interactions. Mittal et al. [32] noted lower OAC for PF (1.36).

Interestingly, the hydrophilic–lipophilic index (HLI) remains relatively stable across wheat-based blends (ranging from 1.75 to 1.80) but shows a marked increase to 4.98 in pure PF, indicating a significant shift in the balance between hydrophilic and lipophilic properties. The statistical analysis, indicated by lower-case letters, confirms significant differences between samples for most parameters, particularly at higher PF concentrations, underlining the substantial impact of PF addition on the techno-functional properties of these binary blends.

Pasting characteristics of PF/soft wheat blends are presented in Table 3.

As PF content increased, the pasting parameters revealed significant gelatinisation and retrogradation changes. In the control sample (pure SWF), the characteristic values typical for wheat-based systems were observed: a peak viscosity of 2444.0 mPa·s, trough viscosity of 1386.5 mPa·s, and final viscosity of 2920.0 mPa·s. These baseline values reflected SWF’s normal starch gelatinisation behaviour, with its characteristic swelling and subsequent reorganization during the cooling phase.

As PF incorporation increased from 5% to 20%, a systematic decrease in peak viscosity was observed from 2342.5 mPa·s to 1859.5 mPa·s, indicating a reduced capacity for starch granule swelling during heating. This trend was accompanied by a corresponding decrease in trough viscosity from 1273.0 mPa·s to 901.0 mPa·s, suggesting diminished stability of the swollen granules under continued heating and shear. The breakdown values, representing the difference between peak and trough viscosities, show minor variations across the blends, ranging from approximately 958.5 mPa·s to 1072.5 mPa·s, indicating similar granule fragility despite compositional changes. Aljahani (2022) [25] observed similar trends in wheat/yellow pumpkin composite flours.

Final viscosity values demonstrated a declining trend from 2920.0 mPa·s in the control sample to 2000.5 mPa·s at 20% PF addition, suggesting reduced starch retrogradation during cooling. The setback values, indicating the degree of retrogradation, showed a gradual decrease from 1533.5 mPa·s to 1099.5 mPa·s with increasing PF content. Similar trends were observed in the research of Aljahani (2022) [25]. Interestingly, the pasting temperature remained relatively stable across all blends (around 87 °C), while peak time decreased slightly from 6.42 to 5.64 s. Promsakha na Sakon Nakhon et al. [35] reported that the protein and lipid fractions in PF interact with amylose, reducing viscosity values. This may also be attributed to the reduction in starch content of the flour, lower swelling rate, and higher fibres as the proportion of PF increases [30].

**Figure 1:**
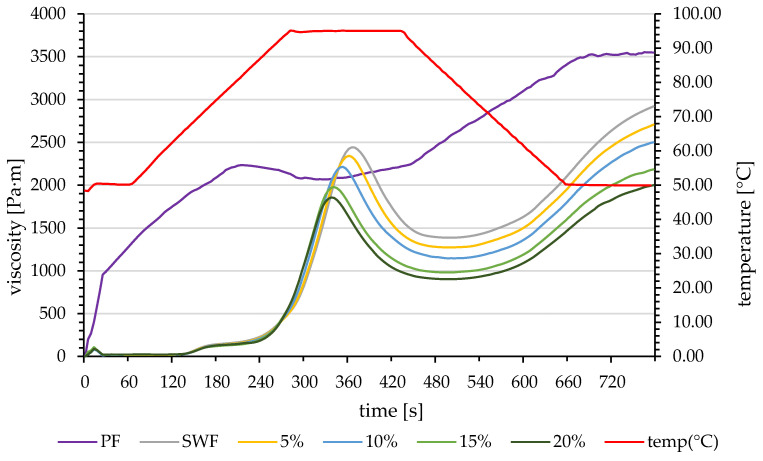
Pasting profiles of pumpkin flour/soft wheat blends; SWF—soft wheat flour; PF—pumpkin flour; 5–20%—pumpkin flour addition.

Pure PF exhibited distinctly different pasting behaviour, with a peak viscosity of 2265.0 mPa·s, an unusually high trough viscosity of 2098.0 mPa·s, and consequently a very low breakdown value of 167 mPa·s. This suggests a highly stable gel structure during continued heating. Pure PF’s notably lower pasting temperature (50.2 °C) and high final viscosity (3549.5 mPa·s) indicate fundamentally different gelatinization mechanisms compared to wheat-based systems, resulting from high fibre, especially insoluble fibre content. The pasting temperature noted by Mardiah et al. [36] and Slamet et al. [37] was similar, but that by Aktaş and Gerçekaslan [34] for pumpkin pulp flour was much higher (84.00 ± 0.01). The pasting profiles of pumpkin flour/soft wheat blends are shown in Figure 1.

The control sample (pure SWF) showed a typical wheat starch gelatinization profile, with a sharp viscosity increase beginning around 67–73 s as the temperature approached 65–70 °C. This initial rapid viscosity development led to a well-defined peak viscosity of approximately 2500 mPa·s, reflecting maximum starch granule swelling. Several systematic changes were observed as PF concentration increased from 5% to 20%. There was a progressive reduction in peak viscosity, with each increment of PF resulting in lower maximum viscosity values. The onset of gelatinization was slightly delayed, particularly noticeable at higher PF concentrations. The samples also showed reduced holding strength during the constant-temperature phase (95 °C) and lower final viscosities during the cooling phase, indicating reduced retrogradation tendency.

The pure PF sample exhibited markedly different behaviour, characterized by an earlier onset of viscosity development and a more gradual viscosity increase than the sharp peak seen in wheat-based samples. It shows higher stability during the holding period with minimal breakdown and a substantial viscosity increase during cooling, suggesting different network formation mechanisms. These pasting profiles provide visual confirmation of the numerical data presented in Table 3 and demonstrate how PF incorporation fundamentally alters the gelatinization and retrogradation behaviour of the blends, likely due to interference with normal starch swelling and network formation processes by the additional fibre and protein components contributed by PF.

A previous study [14] showed similar results, indicating that peak viscosity is affected by adding PF due to its starch content and water absorption. Since a higher quantity of PF can alter peak viscosity, it will also contribute to the ability of the dough to hold moisture, increasing its quality. PF can change the trough viscosity, creating a more stable dough that better holds a shape during baking [38]. Setback viscosity may affect product shelf life and freshness, depending on the extent of change in setback viscosity caused by PF incorporation [39].

The texture parameters of gels obtained from heated paste made of pumpkin flour/soft wheat blends are presented in Table 4.

The control sample of pure SWF demonstrated characteristic textural parameters typical for wheat-based gels. It exhibits the expected viscoelastic behaviour dominated by gelatinised wheat starch and protein interactions with a hardness value of 0.46 N, cohesiveness of 0.815, and springiness of 0.842. The gumminess (0.312 N) and resilience (0.576) values further confirmed the balanced textural profile of the SWF gel, reflecting its capacity to maintain structural integrity under deformation. These observations align with previously reported characteristics of wheat gels, where the starch–protein network contributes to cohesive and elastic properties [25,40].

Gel texture characteristics were systematically modified as the PF concentration increased from 5% to 20%. Hardness consistently decreased from 0.44 N at 5% incorporation to 0.35 N at 20%, indicating progressively softer gel structures. This reduction in hardness was consistent with studies by Batista et al. (2018) [25], which associated higher moisture retention and fibre content in PF with a softer texture due to interference in the starch–protein matrix. This trend is accompanied by a more substantial decline in cohesiveness, dropping from 0.777 at 5% to 0.378 at 20% PF content, suggesting reduced internal binding strength within the gel matrix, likely due to its non-starch polysaccharides affecting gel integrity [41].

The significant decrease in springiness, from 0.792 to 0.123 with increasing PF content, indicated diminished elastic recovery. This trend suggests dilution of the gluten network, reducing the gel’s ability to regain its shape after deformation [25,42]. Interestingly, resilience values remained relatively stable across wheat-based blends, ranging from 0.419 to 0.549, suggesting maintained instantaneous elastic recovery despite other textural changes. The reduction in gumminess from 0.272 N at 5% to 0.014 N at 20% reflects the combined effect of decreased hardness and cohesiveness. This aligns with Batista et al.’s (2018) [25] findings, which reported lower gumminess in PF-enriched systems, possibly contributing to a softer texture.

The textural profile of pure PF gel differed significantly, with minimal hardness (0.04 N), and zero cohesiveness and springiness, resulting in zero gumminess. However, it exhibited a higher resilience (1.251), which may be attributed to the unique properties of pumpkin polysaccharides that influence gel structure formation [25,42]. The functional properties of dough are impacted by the type of SWF used and the variety of other ingredients, such as PF. Research has shown that PF can improve the water retention capacity of the dough, which is fundamental in reaching both the texture and the consistency required of the baked product [13,43]. This higher degree of water retention may extend the dough development time, which might interfere with the baking procedure and the overall quality of the product [38].

The research noted that increasing the percentage of PF in the blend can lead to variations in hardness, often resulting in a softer texture due to the higher moisture retention and fibre content of PF [28]. Incorporating PF has been shown to reduce the hardness of baked products, making them more palatable. Studies have shown that doughs with a certain level of PF blended into them have a lower tendency of gumminess values, which can help improve the mouth feel of the product [25].

**Figure 2:**
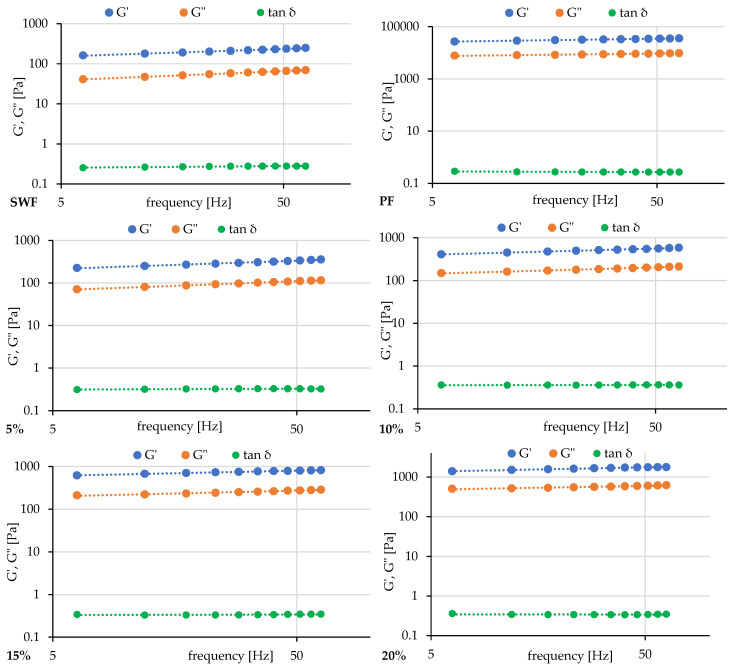
Frequency sweep profiles of pumpkin flour/soft wheat blends. SWF—soft wheat flour; PF—pumpkin flour; 5–20%—pumpkin flour addition.

The frequency sweep profiles of pumpkin flour/soft wheat blends and rheological parameters of gels are shown in Figure 2 and Table 5 accordingly.

The rheological parameters presented in Table 5 provide a detailed understanding of the development of the viscoelastic properties of SWF-PF blends. The control sample exhibited a storage modulus (G′) of 112.7 Pa and a loss modulus (G″) of 26.7 Pa, with a tan δ of 0.2379, indicating that it is primarily elastic. The power law exponents ‘a’ (0.1876) and ‘b’ (0.2298) reflect moderate frequency dependence, while the high G′/G″ ratio (182.3 Pa) confirms its gel-like characteristics, consistent with findings that SWF gels demonstrate viscoelastic solid behaviour due to gelatinized starch and protein interactions.

The incorporation of PF altered the viscoelastic properties significantly. With increasing PF content (from 5% to 20%), there was an apparent increase in both G′ (from 154.7 Pa to 1151.0 Pa) and G″ (from 47.5 Pa to 412.6 Pa), indicating that the gel structure became stronger. The reduction in power law exponents ‘a’ (from 0.1969 Pa to 0.1071 Pa) and ‘b’ (from 0.2150 Pa to 0.0939 Pa) reflects reduced frequency dependence, implying enhanced stability of the gel network. This trend is consistent with previous studies suggesting that adding fibre-rich components like PF can reinforce gel structures by modifying the interactions within the network [42,43].

Despite increasing moduli, the stable tan δ values (0.3064–0.3582) observed across the blends indicate that the gels retain a balanced viscoelastic character even as the structure strengthens. The gradual decrease in the G′/G″ ratio, from 182.3 Pa in the control sample to 103.151 Pa in pure PF, suggests an increase in viscous behaviour as PF content rose, aligning with findings by Ma et al. (2019) [44], who noted a shift towards more viscous properties with PF addition. This shift likely reflects PF’s higher water absorption capacity due to its unique fibre composition, which influences the hydration and swelling of starch granules [45].

Frequency sweep profiles (Figure 2) further validate the numerical data, where the increasing separation between G′ and G″ curves with higher PF levels reflects strengthened gel networks. The decreasing slope of these curves indicates improved structural stability, suggesting that PF incorporation reduces the sensitivity of the gels to frequency changes. The frequency independence observed in pure PF gels, characterized by nearly horizontal curves, indicates a highly stable network, likely due to the polysaccharide content of PF enhancing its gel-forming capacity [46]. The combined analysis of Table 5 and Figure 2 reveals that PF incorporation progressively strengthens the gel structure while enhancing its stability against frequency changes. This behaviour likely results from the unique composition of PF, particularly its fibre content and protein characteristics, which contribute to a more robust network formation. These rheological changes significantly affect product texture and processing considerations in food applications.

The study by Ma et al. (2019) showed similar outcomes, with a decrease in the G′/G″ ratio indicating a change towards more viscous behaviour when PF was substituted for SWF [44]. Since texture is a crucial component of baked products like cakes and cookies, this change has been shown to affect their structural integrity and texture. According to Ma et al. (2019), adding PF at 5% and 25% levels significantly altered the dough’s rheological characteristics [44].

The colour parameter characteristics of pumpkin flour/soft wheat blends are presented in Table 6.

The control sample (pure SWF) exhibited colour parameters typical for refined wheat flour, with a high lightness value (L*) of 91.00, indicating its characteristic bright white appearance. The low a* value (1.03) suggests minimal red colouration, while the b* value of 10.13 indicates a slight yellow tint, typical for wheat flour, due to residual carotenoid content. The resulting chroma (C*) value of 10.18 confirms the overall low colour intensity, while the high hue angle (hº) of 84.3 indicates a predominantly yellow rather than red hue. These observations align with findings in previous studies that note that wheat flour’s pale colour is primarily due to its lower pigment content [40].

As PF was gradually incorporated, there were systematic shifts in the colour attributes of the blends. The decrease in L* value (from 89.23 to 84.28) with increasing PF content indicates a darkening effect, which can be linked to the pigments inherent in pumpkins, such as carotenoids. The increase in both a* (from 1.98 to 5.68) and b* (from 14.28 to 27.13) values suggests a more pronounced red and yellow hue, respectively, consistent with the natural pigmentation of pumpkin [38]. These changes increased chroma (from 14.41 to 27.71), reflecting enhanced colour saturation and intensity. The gradual reduction in hue angle (from 82.14 to 78.18) as PF content rose indicates a shift toward a more reddish-yellow hue.

The pure PF sample stands out with significantly lower L* (69.50), and much higher a* (16.05) and b* (43.50) values, leading to a high chroma (46.37) and lower hue angle (69.71). This distinct colour profile aligns with the literature, which attributes the intense colour of PF to its carotenoid content, specifically β-carotene, which imparts a rich yellow-orange hue [34,38]. The substantial reduction in lightness and shift toward deeper red and yellow tones are consistent with studies reporting similar trends when PF is added to wheat-based products [34].

### 3.2. Bioactive Characteristics of Pumpkin Flour/Soft Wheat Blends

Obtained samples of composite flours were analyzed to characterize the bioactive profile. The reducing sugar content of blends is shown in Table 7.

The control sample (pure soft SWF) exhibited a glucose equivalent value of 2.60 ± 0.18 mg/1 g dry matter, showing the natural reducing sugar content typical for wheat flour, primarily resulting from residual maltose and glucose present after grain maturation and processing. This value serves as a reference point for understanding the impact of PF incorporation on the reducing sugar profile of the blends.

As PF concentration increased from 5% to 20%, a consistent and statistically significant increase in reducing sugar content was observed. At 5% incorporation, the GE value rose to 3.51 ± 0.14 mg/1 g DM, showing an immediate impact of PF addition. This trend continued progressively, with values of 4.08 ± 0.09, 5.13 ± 0.07, and 5.85 ± 0.05 mg/1 g DM for 10%, 15%, and 20% PF incorporation, respectively. Pure PF demonstrated the highest reducing sugar content with a GE value of 8.55 ± 0.03 mg/1 g DM, more than three times that of the control SWF. This substantially higher value explains the linear increase observed in the blends. It reflects the inherent carbohydrate composition of pumpkin, including its natural sugars and potentially partially hydrolyzed polysaccharides resulting from the drying process during flour production. This systematic increase in reducing sugars has essential implications for the final products’ technological functionality and nutritional properties, potentially affecting fermentation behaviour, Maillard reaction development, and sweetness perception.

Other studies have shown similar findings, indicating that the natural sugars in PF—particularly reducing sugars—contribute to the overall sweetness of baked products. Tedom et al. (2019) showed the opposite, as in their study, the PF used considerably reduced product sugar content [41]. This demonstrated that the variety and maturity of the pumpkin used intensely impact the resulting content and performance.

The bioactive content of composite flours is presented in Table 8.

The control sample demonstrated baseline values characteristic of wheat flour, with TPC values of 0.19 GAE mg/1 g DM and 0.05 GAE mg/1 g DM for water and ethanol extracts, respectively. Its DPPH-measured antioxidant activity presented values of 76.2 TE mg/1 g DM and 205.8 TE mg/1 g DM for water and ethanol extracts, while ABTS values registered at 3399.8 TE mg/1 g DM and 754.8 TE mg/1 g DM. The oxidative–reducing potential (FRAP) shows values of 0.87 FeSO_4_ mM/1 g DM and 5.87 FeSO_4_ mM/1 g DM for water and ethanol extracts, respectively, establishing reference points for comparing the impact of PF incorporation.

As PF concentration increased from 5% to 20%, we observed systematic enhancements across all bioactive parameters. The TPC values showed consistent increases in both extracts, reaching 0.57 GAE mg/1 g DM and 0.34 GAE mg/1 g DM for water and ethanol extracts, respectively, at 20% incorporation. This trend was mirrored in antioxidant activity, with DPPH values rising to 237.6 TE mg/1 g DM and 557.6 TE mg/1 g DM and ABTS values increasing to 5158.6 TE mg/1 g DM and 1389.5 TE mg/1 g DM for water and ethanol extracts, respectively, at 20% incorporation. The oxidative–reducing potential (FRAP) showed similar enhancement, reaching 3.69 FeSO_4_ mM/1 g DM and 7.69 FeSO_4_ mM at 20% PF addition.

Pure PF exhibited markedly higher values across all parameters, with TPC reaching 1.22 GAE mg/1 g DM and 0.98 GAE mg/1 g DM, DPPH values of 752.9 TE mg/1 g DM and 1042.7 TE mg/1 g DM, and ABTS values of 7685.1 TE mg/1 g DM and 2484.4 TE mg/1 g DM for water and ethanol extracts, respectively. The oxidative–reducing potential showed a substantial increase to 20.24 FeSO_4_ mM/1 g DM and 32.42 FeSO_4_ mM/1 g DM, demonstrating the significant bioactive potential of pure PF.

A two-way analysis of variance (ANOVA) was conducted to investigate the effect of two factors—the type of extractant and the amount of added pumpkin raw material—on SWF’s antioxidant and reducing properties and total polyphenol content. The results reveal significant differences across all parameters, and notably, water extracts generally show higher values for TPC and ABTS measurements. In contrast, ethanol extracts demonstrate superior DPPH and oxidative–reducing potential, suggesting that each solvent preferentially extracts different compounds. The ABTS-measured antioxidant activity of SWF samples (which measures the quenching reaction of the cation radical ABTS•+ by antioxidants) significantly depended on the type of extractant used and was 4.5 times higher when ethanol extraction was used. Furthermore, for both aqueous and ethanol extraction, all variants of the raw material tested showed significantly higher antioxidant activity than the control raw material.

Ke-Xue et al. similarly confirmed that wheat germ’s aqueous extract showed lower ABTS•+ cation radical scavenging activity than ethanol extract [47]. The same effect was observed for the DPPH method, 2.7 times higher for the alcohol extract than for the aqueous extract. Furthermore, that study observed that the anti-free radical activity increased with increasing ethanol concentration in the extract and was highest for the 70% ethanol extract [47]. Adding PF resulted in a significant increase in the antioxidant activity value of blends (DPPH method) for both the ethanol extract and the water extract (where a higher increase was observed). Partly different results were obtained in a study by Pinna et al., where the highest value of antioxidant properties was confirmed for pumpkin aqueous–alcohol extracts with a percentage of ethanol not exceeding 50% [48].

The amount of PF addition significantly increased the reducing activity (FRAP) values of the test samples in ethanol extracts. In the case of aqueous extraction, variants of the tested raw material with 15% and 20% PF content showed a significant increase in reducing activity. The opposite results were obtained by Pinna et al., who found that <50% ethanol–water extracts of pumpkin (varieties Hokkaido, Lunga di Napoli, Mantovana, Moscata di Provenza, and Violina rugosa) showed lower reducing activity than extracts prepared using 80:20 (*v*/*v*) methanol–water [48]. This may have been because bioactive properties, including reducing properties, change depending on the variety tested, the fertilization regime, and the season (year) in which the pumpkin variety was grown [49].

The content of polyphenolic compounds extracted from SWF measured by the Folin–Ciocâlteu reagent method significantly depended on the type of extractant used. For both aqueous and alcohol extraction, all variants of the raw material tested showed a significantly higher content of polyphenolic compounds than the control raw material. This can be explained by the different ability of phenolic compounds to form complexes with carbohydrates and proteins, which are extracted in water and ethanol to varying degrees, depending on the cereal variety studied [50]. In addition, our results indicate that the polyphenol content of the raw pumpkin was significantly higher in the aqueous extract than in the ethanol extract. The obtained effect is probably because, as research indicates, gallic acid and chlorogenic acid are the most abundant phenolic compounds in pumpkin flesh. These compounds are polar and thus highly soluble in alcohol and even more soluble in water [51]. It should be noted that increasing the amount of PF added significantly affected the total polyphenol content of the extracts tested. It would probably be possible to perform an effective extraction even at amounts higher than 20% (pumpkin pulp powder). Also, in a study by Kulczyński et al., it was found that aqueous extracts from the flesh of the Yellow Melon variety showed a higher total content of polyphenolic compounds (255.69 mg GAE/100 g dw) than water–alcohol extracts (methanol–water, 80:20, *v*/*v*) (232.5 mg GAE/100 g dw) [52]. The increasing total content of polyphenolic compounds in the tested sample (due to the addition of PF) may also be due to phenolic acids such as syringic acid, which can be present in pumpkin in a wide range of amounts of 0.44–6.61 mg/100 g FW [53,54]. Adding PF to SWF blends raised the overall polyphenol content. It improved the antioxidant activity of the final products. Liubych et al. (2023) showed that blends with more significant amounts of PF showed improved DPPH scavenging activity, suggesting better antioxidant potential [55]. In contrast, it was observed that PF significantly improved the blend’s antioxidant characteristics [55].

This comprehensive analysis reveals that PF incorporation not only enhances the bioactive profile of the blends in a concentration-dependent manner but also demonstrates different extraction efficiencies based on solvent polarity. These findings have important implications for both the nutritional value and potential health benefits of products incorporating these blends, while also providing insights into optimal extraction conditions for maximizing bioactive compound recovery.

The interrelation between colour development, reduced sugar content, and bioactive properties has significant implications for processing conditions and final product characteristics. It suggests that visual assessment could serve as a preliminary indicator of bioactive compound content, while reducing sugar levels might need to be considered in processing parameters to optimize sensory attributes and nutritional benefits.

## 4. Conclusions

The systematic investigation of PF incorporation into SWF demonstrated multifaceted effects on technological functionality and nutritional properties. Higher proportions of fine particles in PF significantly influenced water interactions and functional properties. While increased water absorption capacity benefits dough development and bread freshness, pasting behaviour and gluten network formation modifications necessitate careful optimization for bread applications.

Rheological and textural analyses indicated optimal incorporation levels of 5–10% for bread formulation. These concentrations maintain adequate viscoelastic properties while enhancing nutritional value through increased bioactive compounds and antioxidant activity. Progressive weakening of gel structure and reduced pasting viscosities at 15–20% incorporation levels may compromise bread volume and crumb structure. However, these concentrations could be suitable for products where extensive gluten network development is less critical, such as cookies or muffins.

Colour changes induced by PF addition could benefit whole-grain-type products where consumers value darker crumb colour. Increased reduced sugar content and enhanced antioxidant properties suggest benefits for fermentation activity and product shelf life while contributing to nutritional value.

The contrasting behaviour of water- and ethanol-soluble bioactive compounds indicates a need for controlled processing conditions to maximize beneficial compound retention. Stable increases in antioxidant activity across both solvent systems suggest robust enhancement of nutritional value post thermal processing.

This study demonstrates the successful enhancement of wheat-based products’ nutritional profiles through PF incorporation while maintaining acceptable technological functionality at appropriate incorporation levels. These findings advance the understanding of functional ingredient incorporation in cereal-based products and provide practical guidelines for bakery industry product development.

## Figures and Tables

**Table 1:** Granulometry distribution of raw materials.

Mesh Size	SWF	PF
[µm]	[%]	[%]
>200	72.96 ± 10.94 ^d^	60.76 ± 9.11 ^c^
200< >180	6.70 ± 1.01 ^c^	3.78 ± 0.57 ^a^
180< >150	7.74 ± 1.16 ^c^	5.30 ± 0.80 ^a^
150< >125	4.28 ± 0.64 ^b^	4.86 ± 0.73 ^a^
125< >106	4.00 ± 0.60 ^b^	4.94 ± 0.74 ^a^
106< >80	3.86 ± 0.58 ^b^	4.60 ± 0.69 ^a^
<80	1.10 ± 0.17 ^a^	13.26 ± 1.99 ^b^

SWF—soft wheat flour; PF—pumpkin flour; control lower-case letters mean values in columns are statistically different (*p* = 0.05).

**Table 2:** Techno-functional characteristics of the pumpkin flour/soft wheat binary blends.

Sample	WHC	WAC	WAI	SP	WSI	OAC	HLI
	g H_2_O/g DM				g H_2_O/100 g DM	g Oil/g DM	
SFW	2.52 ± 0.00 ^a^	1.83 ± 0.03 ^a^	5.62 ± 0.03 ^a^	5.84 ± 0.01 ^a^	3.80 ± 0.36 ^a^	1.14 ± 0.02 ^a^	1.80 ± 0.00 ^a^
5%	2.52 ± 0.02 ^a^	1.90 ± 0.07 ^ab^	5.68 ± 0.10 ^a^	5.91 ± 0.15 ^a^	4.62 ± 0.14 ^ab^	1.18 ± 0.02 ^b^	1.79 ± 0.01 ^a^
10%	2.78 ± 0.11 ^b^	2.04 ± 0.07 ^ab^	5.80 ± 0.03 ^ab^	6.15 ± 0.03 ^ab^	5.82 ± 0.06 ^ab^	1.26 ± 0.01 ^c^	1.79 ± 0.03 ^a^
15%	3.07 ± 0.04 ^c^	2.14 ± 0.00 ^ab^	5.94 ± 0.07 ^b^	6.38 ± 0.00 ^b^	6.84 ± 1.10 ^bc^	1.33 ± 0.00 ^d^	1.78 ± 0.02 ^a^
20%	3.56 ± 0.15 ^d^	2.24 ± 0.04 ^b^	6.88 ± 0.15 ^c^	7.53 ± 0.16 ^c^	8.60 ± 0.00 ^c^	1.41 ± 0.01 ^e^	1.75 ± 0.03 ^a^
PF	10.89 ± 0.14 ^e^	8.01 ± 0.33 ^c^	8.64 ± 0.14 ^d^	12.22 ± 0.23 ^d^	29.27 ± 2.47 ^d^	1.78 ± 0.02 ^f^	4.98 ± 0.04 ^b^

SWF—soft wheat flour; PF—pumpkin flour; 5–20%—pumpkin flour addition; WHC—water-holding capacity; WAC—water absorption capacity; OAC—oil absorption capacity; HLI—hydrophilic/lipophilic index; WAI—water absorption index; WSI—water solubility index; SP—swelling power; lower-case letters mean significant differences in columns at *p* = 0.05.

**Table 3:** Pasting parameters of pumpkin flour/soft wheat blends.

Sample	Peak Viscosity [mPa*·*s]	Trough Viscosity [mPa*·*s]	Breakdown [mPa*·*s]	Final Viscosity [mPa*·*s]	Setback [mPa*·*s]	Pasting Temp [°C]	Peak Time [s]
SWF	2444.0 ± 4.2 ^e^	1386.5 ± 0.7 ^d^	1057.5 ± 3.5 ^c^	2920.0 ± 12.7 ^c^	1533.5 ± 13.4 ^d^	87.6 ± 0.53 ^b^	6.42 ± 0.40 ^a^
5%	2342.5 ± 27.6 ^de^	1273.0 ± 1.4 ^c^	1069.5 ± 29.0 ^c^	2706.0 ± 26.9 ^bc^	1433.0 ± 28.3 ^cd^	87.7 ± 0.57 ^b^	6.00 ± 0.00 ^a^
10%	2219.0 ± 25.5 ^cd^	1146.5 ± 12.0 ^b^	1072.5 ± 13.4 ^c^	2501.0 ± 38.2 ^b^	1354.5 ± 26.2 ^bc^	87.5 ± 0.48 ^b^	5.40 ± 0.57 ^a^
15%	1978.5 ± 92.6 ^b^	981.0 ± 46.7 ^a^	997.5 ± 46.0 ^bc^	2184.0 ± 84.9 ^a^	1203.0 ± 38.2 ^ab^	87.2 ± 0.04 ^b^	5.67 ± 0.00 ^a^
20%	1859.5 ± 13.4 ^a^	901.0 ± 4.2 ^a^	958.5 ± 9.2 ^c^	2000.5 ± 36.1 ^a^	1099.5 ± 31.8 ^ab^	87.3 ± 0.04 ^b^	5.64 ± 0.05 ^a^
PF	2265.0 ± 14.1 ^cd^	2098.0 ± 89.1 ^e^	167 ± 75.0 ^a^	3549.5 ± 231.2 ^d^	1451.5 ± 142.1 ^cd^	50.2 ± 0.00 ^a^	5.17 ± 2.60 ^a^

SWF—soft wheat flour; PF—pumpkin flour; 5–20%—pumpkin flour addition; lower-case letters mean values in columns are statistically different (*p* = 0.05).

**Table 4:** Texture parameters of gels made from pumpkin flour/soft wheat blends.

Sample	Hardness [N]	Cohesiveness	Springiness	Gumminess [N]	Resilience
SWF	0.46 ± 0.01 ^c^	0.815 ± 0.04 ^c^	0.842 ± 0.01 ^e^	0.312 ± 0.01 ^c^	0.576 ± 0.02 ^a^
5%	0.44 ± 0.01 ^c^	0.777 ± 0.00 ^c^	0.792 ± 0.00 ^d^	0.272 ± 0.01 ^b^	0.507 ± 0.02 ^a^
10%	0.40 ± 0.06 ^bc^	0.778 ± 0.02 ^c^	0.763 ± 0.02 ^d^	0.236 ± 0.04 ^b^	0.549 ± 0.02 ^a^
15%	0.34 ± 0.04 ^b^	0.482 ± 0.10 ^b^	0.165 ± 0.03 ^c^	0.028 ± 0.01 ^a^	0.419 ± 0.10 ^a^
20%	0.35 ± 0.02 ^b^	0.378 ± 0.02 ^b^	0.123 ± 0.00 ^b^	0.014 ± 0.00 ^a^	0.452 ± 0.09 ^a^
PF	0.04 ± 0.00 ^a^	0.000 ± 0.00 ^a^	0.000 ± 0.00 ^a^	0.000 ± 0.00 ^a^	1.251 ± 0.15 ^b^

SWF—soft wheat flour; PF—pumpkin flour; 5–20%—pumpkin flour addition; lower-case letters mean values in columns are statistically different (*p* = 0.05).

**Table 5:** Rheological properties of pumpkin flour/soft wheat blends.

Sample	G′ [Pa]	a	G″ [Pa]	b	tan δ	c	G′ = G″ [Pa]
SWF	112.7 ± 11.5 ^a^	0.1876 ± 0.0151 ^c^	26.7 ± 1.6 ^a^	0.2298 ± 0.0125 ^c^	0.2379 ± 0.0103 ^a^	0.0418 ± 0.0025 ^d^	182.3 ± 5.3 ^d^
5%	154.7 ± 5.4 ^a^	0.1969 ± 0.0107 ^c^	47.5 ± 8.4 ^a^	0.2150 ± 0.0100 ^c^	0.3064 ± 0.0427 ^bc^	0.0184 ± 0.0199 ^c^	160.2 ± 1.3 ^c^
10%	309.0 ± 15.9 ^b^	0.1515 ± 0.0054 ^b^	109.7 ± 2.1 ^b^	0.1570 ± 0.0008 ^b^	0.3556 ± 0.0247 ^bc^	0.0055 ± 0.0045 ^bc^	122.7 ± 10.8 ^b^
15%	497.4 ± 22.5 ^c^	0.1215 ± 0.0001 ^a^	160.1 ± 14.7 ^b^	0.1365 ± 0.0088 ^b^	0.3215 ± 0.0154 ^c^	0.0149 ± 0.0093 ^c^	96.3 ± 16.4 ^ab^
20%	1151.0 ± 84.6 ^d^	0.1071 ± 0.0025 ^a^	412.6 ± 47.9 ^c^	0.0939 ± 0.0017 ^a^	0.3582 ± 0.0153 ^c^	−0.0130 ± 0.0042 ^ab^	120.5 ± 1.3 ^b^
PF	21,570.0 ± 1576.9 ^e^	0.1205 ± 0.0024 ^a^	6362.1 ± 445.6 ^d^	0.0979 ± 0.0052 ^a^	0.2949 ± 0.0001 ^b^	−0.0225 ± 0.0035 ^a^	103.151 ± 6.417 ^ab^

Control—soft wheat flour (SWF); PF—pumpkin flour; 5–20%—pumpkin flour addition; lower-case letters mean values in columns are statistically different (*p* = 0.05).

**Table 6:** Colour parameters of pumpkin flour/soft wheat blends.

Sample	L*	a*	b*	C*	hº
Control	91.00 *±* 0.95 ^d^	1.03 *±* 0.10 ^a^	10.13 *±* 0.62 ^a^	10.18 *±* 0.62 ^a^	84.3 *±* 0.29 ^e^
5%	89.23 *±* 0.59 ^d^	1.98 *±* 0.30 ^b^	14.28 *±* 0.39 ^b^	14.41 *±* 0.42 ^b^	82.14 *±* 0.95 ^d^
10%	87.00 *±* 0.37 ^c^	3.43 *±* 0.31 ^c^	19.98 *±* 0.46 ^c^	20.27 *±* 0.48 ^c^	80.27 *±* 0.79 ^c^
15%	85.55 *±* 0.60 ^bc^	4.00 *±* 0.12 ^d^	22.60 *±* 0.47 ^a^	22.95 *±* 0.48 ^d^	79.96 *±* 0.15 ^c^
20%	84.28 *±* 0.46 ^b^	5.68 *±* 0.30 ^e^	27.13 *±* 0.74 ^e^	27.71 *±* 0.76 ^e^	78.18 *±* 0.51 ^b^
PF	69.50 *±* 3.18 ^a^	16.05 *±* 0.42 ^f^	43.50 *±* 2.64 ^f^	46.37 *±* 2.52 ^f^	69.71 *±* 1.11 ^a^

Control—soft wheat flour; PF—pumpkin flour; 5–20%—pumpkin flour addition; lower-case letters mean values in columns are statistically different (*p* = 0.05).

**Table 7:** Reducing sugar content as glucose equivalent in pumpkin flour/soft wheat blends.

Sample	GE mg/1 g DM
SWF	2.60 ± 0.18 ^a^
5%	3.51 ± 0.14 ^b^
10%	4.08 ± 0.09 ^c^
15%	5.13 ± 0.07 ^d^
20%	5.85 ± 0.05 ^e^
PF	8.55 ± 0.03 ^f^

SWF—soft wheat flour; PF—pumpkin flour; 5–20%—PF addition level; lower-case letters mean values in columns are statistically different (*p* = 0.05).

**Table 8:** Total polyphenol content, antioxidant activity and oxidative–reducing characteristic of pumpkin flour/soft wheat blends.

Sample	TPC (GAE mg/1 g DM)		DPPH (TE mg/1 g DM)		ABTS (TE mg/1 g DM)		FRAP (FeSO_4_ mM/1 g DM)	
	H_2_O	EtOH	H_2_O	EtOH	H_2_O	EtOH	H_2_O	EtOH
SWF	0.19 ± 0.00 ^a^	0.05 ± 0.01 ^a^	76.2 ± 3.3 ^a^	205.8 ± 7.5 ^a^	3399.8 ± 149.1 ^a^	754.8 ± 42.8 ^a^	0.87 ± 0.03 ^a^	5.87 ± 0.12 ^a^
5%	0.28 ± 0.02 ^b^	0.13 ± 0.00 ^b^	110.0 ± 3.5 ^b^	415.6 ± 7.8 ^b^	3927.7 ± 17.1 ^b^	866.0 ± 26.8 ^b^	1.06 ± 0.08 ^a^	6.54 ± 0.02 ^b^
10%	0.42 ± 0.01 ^c^	0.21 ± 0.01 ^c^	166.7 ± 6.9 ^c^	457.7 ± 18.5 ^c^	4451.3 ± 10.6 ^c^	987.5 ± 13.2 ^c^	1.85 ± 0.05 ^a^	6.82 ± 0.24 ^bc^
15%	0.48 ± 0.01 ^d^	0.25 ± 0.01 ^d^	200.3 ± 3.0 ^d^	486.5 ± 1.8 ^c^	4928.3 ± 72.6 ^d^	1176.1 ± 3.1 ^d^	2.98 ± 0.29 ^b^	7.35 ± 0.02 ^cd^
20%	0.57 ± 0.01 ^e^	0.34 ± 0.01 ^e^	237.6 ± 7.5 ^e^	557.6 ± 7.8 ^d^	5158.6 ± 112.6 ^d^	1389.5 ± 27.4 ^e^	3.69 ± 0.14 ^b^	7.69 ± 0.06 ^d^
PF	1.22 ± 0.02 ^f^	0.98 ± 0.03 ^f^	752.9 ± 6.7 ^f^	1042.7 ± 23.8 ^e^	7685.1 ± 136.3 ^e^	2484.4 ± 22.8 ^f^	20.24 ± 1.00 ^c^	32.42 ± 0.51 ^e^
sample	***		***		***		***	
solvent	***		***		***		***	
Sample × solvent	***		***		***		***	

SWF—soft wheat flour; PF—pumpkin flour; 5–20%—pumpkin flour addition; lower-case letters mean values in columns are statistically different (*p* = 0.05). ***—statistically different for *p* > 0.001.

## Data Availability

The original contributions presented in this study are included in the article. Further inquiries can be directed to the corresponding author.
